# Losartan Interactions with 2-Hydroxypropyl-β-CD

**DOI:** 10.3390/molecules27082421

**Published:** 2022-04-08

**Authors:** Vasiliki Palli, Georgios Leonis, Nikoletta Zoupanou, Nikitas Georgiou, Maria Chountoulesi, Nikolaos Naziris, Demeter Tzeli, Costas Demetzos, Georgia Valsami, Konstantinos D. Marousis, Georgios A. Spyroulias, Thomas Mavromoustakos

**Affiliations:** 1 Department of Chemistry, School of Sciences, National and Kapodistrian University of Athens, 15784 Athens, Greece; vassopalli192@gmail.com (V.P.); georgios.leonis@gmail.com (G.L.); nikolzoup@chem.uoa.gr (N.Z.); nikitage@chem.uoa.gr (N.G.); tzeli@chem.uoa.gr (D.T.); 2 Department of Pharmacy, School of Health Sciences, National and Kapodistrian University of Athens, 15784 Athens, Greece; mchountoules@pharm.uoa.gr (M.C.); niknaz@pharm.uoa.gr (N.N.); demetzos@pharm.uoa.gr (C.D.); 3 Department of General Biophysics, Faculty of Biology and Environmental Protection, University of Lodz, Pomorska 141/143, 90-236 Lodz, Poland; 4 Theoretical and Physical Chemistry Institute, National Hellenic Research Foundation, 48 Vassileos Constantinou Ave., 11635 Athens, Greece; 5 Department of Pharmacy, University of Patras, 26504 Patras, Greece; kmarouk@upatras.gr (K.D.M.); g.a.spyroulias@upatras.gr (G.A.S.)

**Keywords:** losartan, hydroxypropyl-β-cyclodextrin, molecular interactions, differential scanning calorimetry, nuclear magnetic resonance, molecular dynamics

## Abstract

Losartan potassium salt (LSR) is a well-known antihypertensive drug with proven beneficial effects on human health. Its formulation with the non-toxic 2-hydroxypropyl-β-cyclodextrin (2-HP-β-CD) could improve its pharmacological profile. Thus, its molecular interactions are studied using a combination of Differential Scanning Calorimetry (DSC), Nuclear Magnetic Resonance (NMR) and Molecular Dynamics (MD). First, its complexation is shown through Differential Scanning Calorimetry as lyophilization provided distinct thermal properties in comparison to the mixture. The complexation is further proved by utilizing the chemical shift changes in the complexation and T1 values. Furthermore, the reversible favorable complexation was shown by MD calculations. Such physical chemical properties provide evidence that this formulation must be further explored through biological experiments.

## 1. Introduction

**Figure 1:**
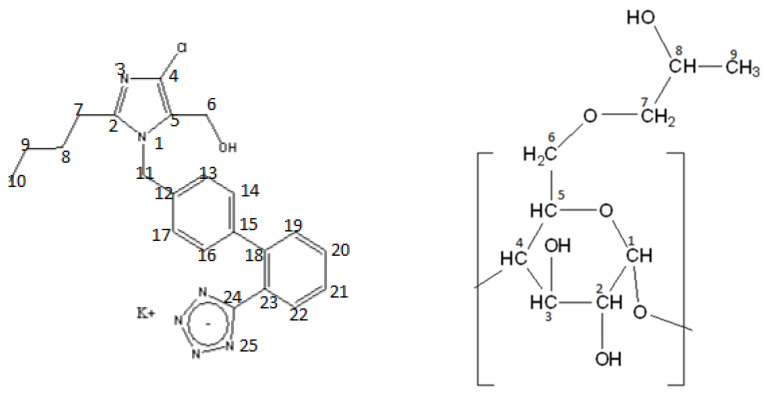
Losartan structure (**left**) and 2-HP-β-CD structure (**right**).

Losartan is a prototype drug (see structure at Figure 1) among AT1 antagonists that acts selectively on the AT1 receptor and blocks the hypertensive effects of the peptide hormone Angiotensin II. It is administered as antihypertensive drug in a water potassium salt formulation (LSR) [1,2]. Recently losartan was suggested as a promising drug against COVID-19 [3]. As its pharmacological profile is not ideal due to its high lipophilicity and non-optimum bioavailability, efforts are being made using new formulations and utilizing nanotechnology progress [2].

In our recent study, A. Chroni et al. reported the encapsulating losartan potassium into amphiphilic block copolymer micelles, utilizing the biocompatible/biodegradable poly(ethylene oxide)-b-poly(caprolactone) (PEO-b-PCL) diblock copolymer. Light scattering and nuclear magnetic resonance (NMR) provided information on micelle structure and polymer–drug interactions. According to dynamic light scattering (DLS) analysis, the PEO-b-PCL micelles and LSR-loaded PEO-b-PCL nanocarriers formed nanostructures in the range of 17–26 nm in aqueous milieu. Attenuated total reflection Fourier transform infrared (ATR-FTIR) and ultraviolet-visible (UV-Vis) measurements confirmed the presence of LSR in the polymeric drug solutions. Nuclear Magnetic Resonance (NMR) results proved the successful encapsulation of LSR into the PEO-b-PCL micelles by analyzing the drug–micelles intermolecular interactions. Specifically, 2D-NOESY experiments clearly evidenced the intermolecular interactions between the biphenyl ring and butyl chain of LSR structure with the methylene signals of PCL [4].

In another study [5], A. Chroni et al. focused on developing highly stable losartan potassium (LSR) polymeric micellar nanocarriers using two novel amphiphilic poly(n-butyl acrylate)-block-poly(oligo(ethylene glycol) methyl ether acrylate) (PnBA-b-POEGA) copolymers. The LSR-loaded PnBA-b-POEGA nanocarriers presented increased size and greater mass nanostructures compared to empty micelles, implying the successful loading of LSR into the inner hydrophobic domains. 2D-NOESY NMR results showed that LSR-loaded PnBAb-POEGA nanocarriers induced strong intermolecular interactions between the biphenyl ring and the butyl chain of LSR with the methylene signals of PnBA. The highest hydrophobicity of the PnBA27-b-POEGA73 micelles contributed to an efficient encapsulation of LSR into the micelles exhibiting a greater value of % enantiomeric excess (EE) compared to PnBA30-b-POEGA70 + 50% LSR nanocarriers. Ultrasound release profiles of LSR showed that a great amount of the encapsulated LSR is strongly attached to both PnBA30-b-POEGA70 and PnBA27-b-POEGA73 micelles [5].

In another study, de Paula et al. [6] utilized 2HP-β-CD (see structure at Figure 1) to host losartan in an attempt to decrease its hydrophilicity and increase its pharmaceutical efficiency. Usually, cyclodextrins are used to modulate the effects of the very lipophilic drug, but this pioneer study had the opposite aim. Indeed, the in vivo results showed that losartan extends its duration of action when is administered as a guest in 2HP-β-CD. This exciting result triggered our interest to study the interactions of losartan with the same cyclodextrin in detail in order to understand, at the molecular level, the interactions between host and guest [6].

In order to achieve this aim, we have used Differential Scanning Calorimetry (DSC) and NMR spectroscopy. Both techniques are widely utilized and are very prosperous in providing information on the drug:CD interactions. These interactions are critical to understanding the way of action of a drug when CD vehicle is used in the formulation [7,8,9].

The ultimate aim of this study is to characterize and optimize the interactions between losartan and the various cyclodextrins to be studied. These studies will follow with some experimental techniques such as titration NMR and fluorescence spectroscopy in order to compare the in silico studies performed with experimental data.

## 2. Results and Discussion

### 2.1. Differential Scanning Calorimetry

**Figure 2:**
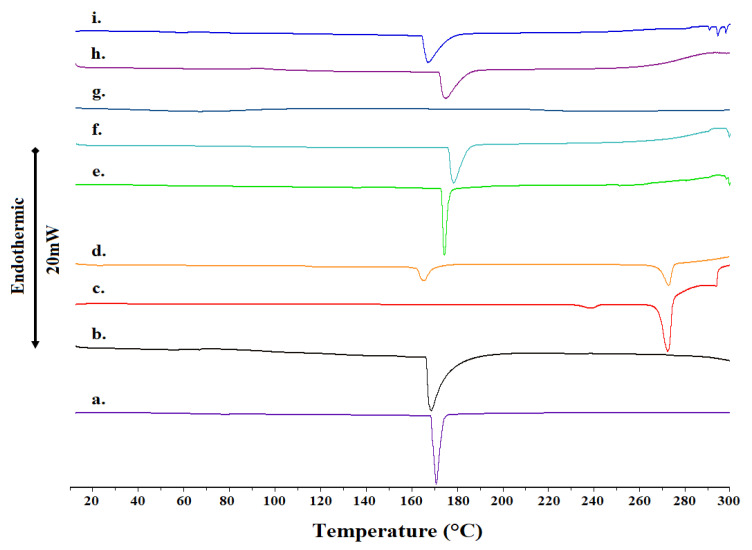
DSC scans of (a.) 2-HP-β-CD raw material; (b.) 2-HP-β-CD lyophilized; (c.) losartan raw material; (d.) losartan lyophilized; (e.) mixture of 2-HP-β-CD and losartan raw materials; (f.) mixture of lyophilized 2-HP-β-CD and raw material losartan; (g.) mixture of raw material 2-HP-β-CD and lyophilized losartan; (h.) mixture of lyophilized 2-HP-β-CD and lyophilized losartan; and (i.) lyophilized complex of 2-HP-β-CD and losartan. The bar represents a heat flow amount of 20 mW.

The complexation of the prototype of AT1R antagonist losartan with 2-HP-β-CD is achieved through lyophilization of the prepared dissolved mixture in a basic aquatic with ammonia solution. It was of interest, then, to study the effect of lyophilization in the prepared complex. Figure 2 shows the thermal scans of nine samples and Table 1 shows their diagnostic parameters. Starting from the bottom sample, the analysis will progress with ascending thermal scans. The bottom sample shows the thermal scan of 2-HP-β-CD in powder form. A narrow peak with maximum at *ca* T_m_ = 171 °C is observed. The endothermic peak of all the CDs corresponds to the characteristic dehydration process of the CD molecules, followed by decomposition taking place above 300 °C [7,8]. The next sample shows the thermal scan of lyophilized HP-β-CD. Again, one peak is observed but at *ca* T_m_ = 169 °C, i.e., two degrees lower than that observed for powder HP-β-CD. In addition, the peak is broader and has less enthalpy, signifying that lyophilization reduces the crystallinity, affecting the thermal properties of a sample. In this case, the lyophilization decreased the phase transition temperature and the enthalpy and broadened the width of the observed peak. This phenomenon may be attributed to the fact that the lyophilized sample of HP-β-CD had already been partially dehydrated by the lyophilization process. In any case, the cyclodextrin samples were in a more crystalline state compared with other studies where HP-β-CD was amorphous [9,10]. A study on M-β-CD documented a sharp transition at 158 °C, similar to ours [11].

The next sample is referred to the thermal scan of a powder losartan. In a powder form it shows two phase transitions, a small broad peak (*ca* T_m_ = 239 °C) that precedes a sharp melting endotherm of high intensity (*ca* Τ_max_ = 272 °C). When losartan is lyophilized, a very different thermal scan is observed. Two broader peaks are observed at different temperatures (*ca* T_m1_ = 165 °C and T_m2_ = 273 °C). It is evident from the observed spectra that lyophilization of losartan results in an increase in the broadening of the peaks and shift of the phase transitions in lower (T_m2_) and higher (T_m1_) values.

In the ascending order, the next thermal scan refers to a powder of 2-HP-β-CD mixed with losartan powder. A sharp peak is observed, centered at *ca* T_m_ = 174 °C. This peak is broadened and moved to a higher temperature when the 2-HP-β-CD used is lyophilized (*ca* T_m_ = 176 °C). The effect was more pronounced when lyophilization was applied to losartan. A very broad peak was observed with no discernable T_m_. When losartan and 2-HP-β-CD are mixed in a lyophilized form, then an even broader peak at *ca* T_m_ = 174 °C is observed. The broadened peak is further shifted to a lower temperature when 2-HP-β-CD and losartan are complexed (*ca* T_m_ = 167 °C). In conclusion, the lyophilized complex form shows a different thermal profile than the lyophilized mixed forms or mixture. In addition, in all cases, the endothermic transitions of losartan were absent, indicating interactions of all drug molecules with the cyclodextrin [12].

A significant observation that is common in all the complexes (Figure 2e–i) is the total disappearance of the drug melting peak for either the raw or the lyophilized form. This is considered to be a strong indication of interaction between the components, evidently attributed to the inclusion of the drug molecule inside the CD cavity. The results suggest strong interaction and a high degree of complexation, which should be confirmed through complementary techniques [7,8,13,14]. Moreover, the total disappearance of the drug melting peak can be directly related to the conversion of the crystalline compound to the amorphous one, which could be the consequence of the formation of a true inclusion complex [8]. Thus, we carried out ^1^H NMR spectroscopy and T1 relaxation experiments in order to further confirm the DSC results and the complexation between losartan and 2-HP-β-CD.

### 2.2. Nuclear Magnetic Resonance

**Figure 3:**
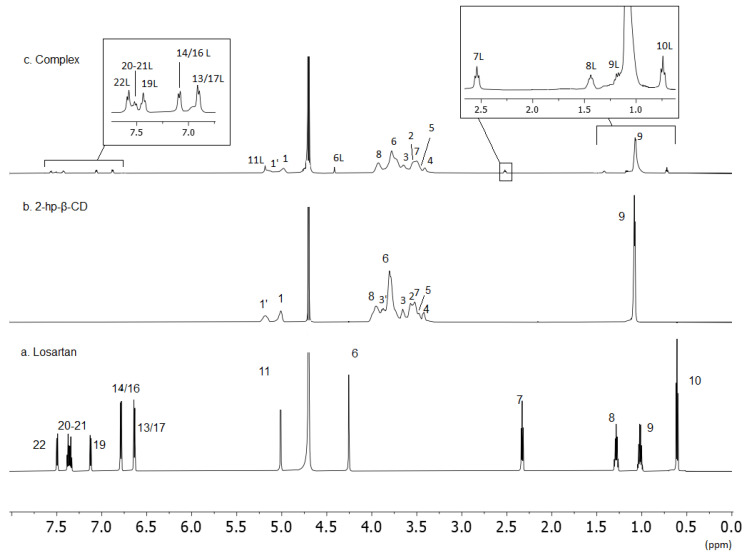
^1^H NMR spectra obtained with 700 MHz Bruker spectrometer using D_2_O solvent and at 25 °C: (**a**) losartan; (**b**) 2-HP-β-CD; (**c**) complex of losartan with 2-HP-β-CD.

One-dimensional and two-dimensional homonuclear (COSY and ROESY) were performed to unambiguously assign proton nuclei of the losartan and 2HP-β-CD in D_2_O. The complex between the two molecules was also assigned with identical experiments. Representative ^1^H NMR spectra of the three molecules with their assignment are shown in Figure 3 (chemical shifts are shown in Table 2).

The chemical shifts of losartan, 2HP-β-CD and the complex are shown in Table 2. From the results in Table 2, the complexation of losartan with 2HP-β-CD is eminent. The aromatic region of losartan in the complex consists of broad peaks shifted towards higher chemical shifts signifying its different environment as it is hosted in the hydrophobic environment of 2HP-β-CD. The same is happening with the alkyl chain. The peaks in the alkyl chain are broadened and again shift downfield. This is indicative that all the molecules of losartan are embedded inside the 2HP-β-CD and does not solely experience the polar environment of deuterated oxide. In conclusion, ^1^H NMR spectroscopy complements results from differential scanning calorimetry and confirms the complexation between losartan and 2HP-β-CD.

**Figure 4:**
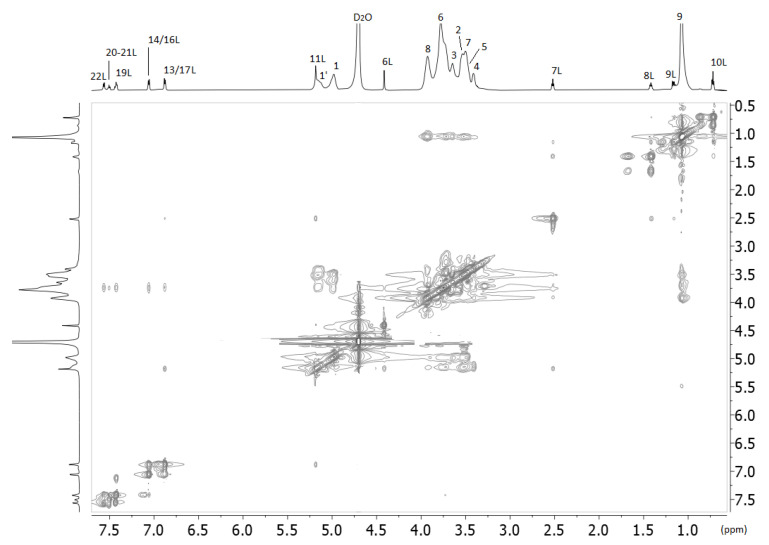
2D ROESY spectra of the losartan and 2-HP-β-CD complex obtained with 700 MHz Bruker spectrometer using D_2_O solvent and at 25 °C.

Another critical experiment that provides valuable information for the complexing is the 2D ROESY experiment shown in Figure 4. Interestingly, all the protons of losartan interact spatially with all the protons of 2HP-β-CD except those that are anomeric. The interactions of the protons of the butyl alkyl chain of losartan as well the H-11 that bridges biphenyl ring with imidazole ring are clearly stronger than the aromatic ones. Spatial interactions are not observed between the methylene H-6 protons of losartan with 2HP-β-CD (Table 3). (See the Supplementary Materials for 2D COSY NMR spectra, Figures S1–S3).

Further strong evidence of the complexation that also gives information about the interactions of the drug molecule with 2HP-β-CD is the T1 relaxation experiments. T1 values of clearly defined protons of 2HP-β-CD in a complex form have reduced values in comparison to the 2HP-β-CD alone dissolved in deuterated water (see Table 4). This is indicative of the complexation as this will reduce the mobility of the 2HP-β-CD protons. In the case of losartan, the picture is more complicated. Aromatic protons show higher T1 values when are complexing rather than when they are in deuterated water solvent. However, the methylenes of the butyl chain are experiencing restricted mobility in the complex form. (See also Supplementary Materials, Figures S4–S6).

**Figure 5:**
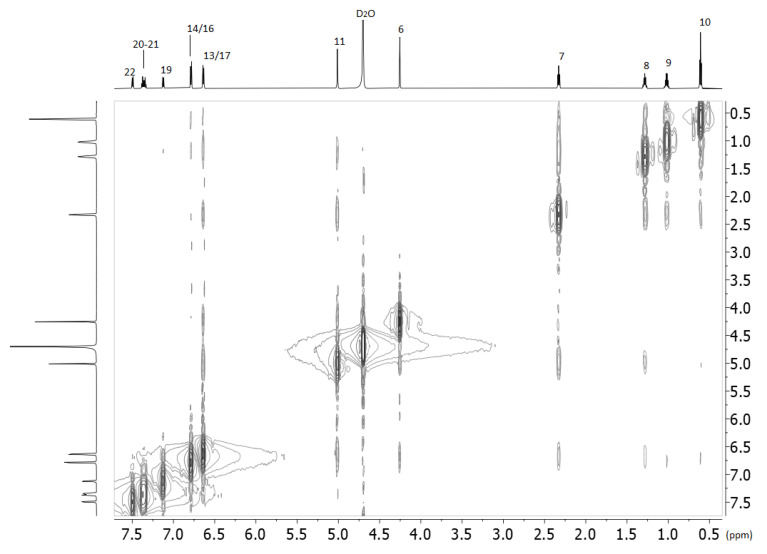
2D ROESY spectra obtained with 700 MHz Bruker spectrometer using D_2_O solvent and at 25 °C of losartan.

An explanation of this result can be given by the 2D ROESY of losartan alone (Figure 5).

Thus, the mobility of the molecule is highly restricted in the deuterated water. This is expected, as losartan is a highly lipophilic molecule (logP = 5.37 ALOGPS and 4.48 with ChemAxon). In the hydrophobic environment of 2HP-β-CD it seems that this restriction is not applied, and losartan appears with higher mobility. (See Supplementary Materials for 2D COSY NMR spectra, Figures S1–S3).

It is evident from this spectrum that the alkyl chain is in spatial relationship with the aromatic rings (see Table 5).

### 2.3. Molecular Dynamics

Lengthy MD calculations for the losartan-2-HP-β-CD complex suggested that the structure of cyclodextrin remains practically stable upon drug complexation throughout the run (simulation time 500 ns). Specifically, root mean square deviations (RMSD) for 2-HP-β-CD fluctuate around an average value of ~2.8 Å (see Supplementary Materials, Figure S7). Similar behavior was observed for losartan, which stabilizes its structure after the first 20 ns of the simulation and moderately deviates from an average of 2.6 Å thereafter; however, larger deviations were observed for the drug at ~250–350 ns of the run, thus suggesting the presence of intermediate conformational changes due to increased flexibility (see Supplementary Materials, Figure S7, right).

**Figure 6:**
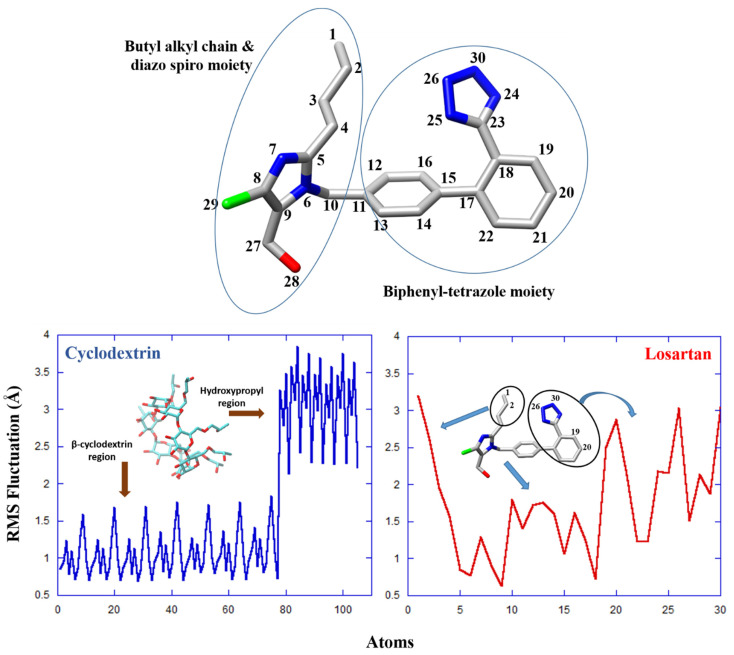
Chemical structure of losartan. Atoms are color coded and numbering refers to the MD calculations. RMSF values for 2-HP-β-CD (blue) and losartan (red) in the drug–cyclodextrin complex. Hydrogen atoms were omitted from the structure and from the calculations.

The structural behavior of the losartan–2-HP-β-CD complex was further investigated though RMS Fluctuation (RMSF) calculations, as presented in Figure 6. It was observed that the β-cyclodextrin segment of 2-HP-β-CD is particularly stable, while the hydroxypropyl groups significantly contribute to the flexibility of the host molecule (Figure 6, blue). Interestingly, the structural variation of losartan during the middle of the simulation, as denoted by the RMSD values above (see Supplementary Materials, Figure S7, right), may be attributed to the flexibility of: (i) the terminal of the butyl alkyl chain (specifically atoms C1, and C2), and (ii) the phenyl-tetrazole group of losartan (atoms N26, N30, C19 and C20) (encircled regions, Figure 6, bottom). We note that while sudden RMSD changes may also indicate significant conformational rearrangements, in this case the aforementioned regions of losartan increase the overall flexibility of the molecule without inducing any permanent structural modifications.

Hydrogen bonding (HB) calculations were in agreement with the DFT results, as they indicated favorable interactions between the hydroxyl or H groups of 2-HP-β-CD and atoms on the tetrazole ring of losartan. It was observed that 2-HP-β-CD forms HBs with N24, N25, and N26 (tetrazole), as well as with N7 in the diazo spiro moiety (Figure 6, top). Apparently, these interactions play an important role in the stabilization of the complex. It was also shown that N30, O28 and N6 (diazo spiro moiety) develop stable HB interactions with water molecules, either independently or through water-bridged HB formation with 2-HP-β-CD. The structural and HB analyses further indicate that specific groups, such as the tetrazole moiety, may enjoy an overall conformational stability (through HBs, e.g., N26), despite any coexisting high atomic fluctuations (e.g., N30).

MM–PBSA and MM–GBSA free-energy calculations identified the main energetic components that determine losartan binding to 2-HP-β-CD. It was shown that complex formation is driven by enthalpy contributions, mainly through van der Waals interactions (Table 6). The electrostatic forces between IRB and 2-HP-β-CD (Δ*E*_elec_) also favor binding; however, the total electrostatics of the system [Δ*G*_elec (tot)_] counteracts the drug–cyclodextrin association due to unfavorable solvent contributions (Δ*G*_PB/GB_). Additional calculation of the entropy term enabled the estimation of the total free energy of binding with the two methods [MM–PB (GB) SA]. In agreement with the DFT-binding energy calculations (Δ*G*_DFT_ = −5.5 kcal/mol), it was suggested that losartan has a sufficient affinity for 2-HP-β-CD (Δ*G*_MM–PBSA_: −4.8 kcal/mol, Δ*G*_MM–GBSA_: −6.7 kcal/mol, Table 1) that allows for drug binding to occur. However, the predicted binding energy may not be adequate to prevent eventual losartan release from its host. Therefore, the ability of 2-HP-β-CD as an effective losartan transporter could be postulated.

### 2.4. Conformational Analysis

**Figure 7:**
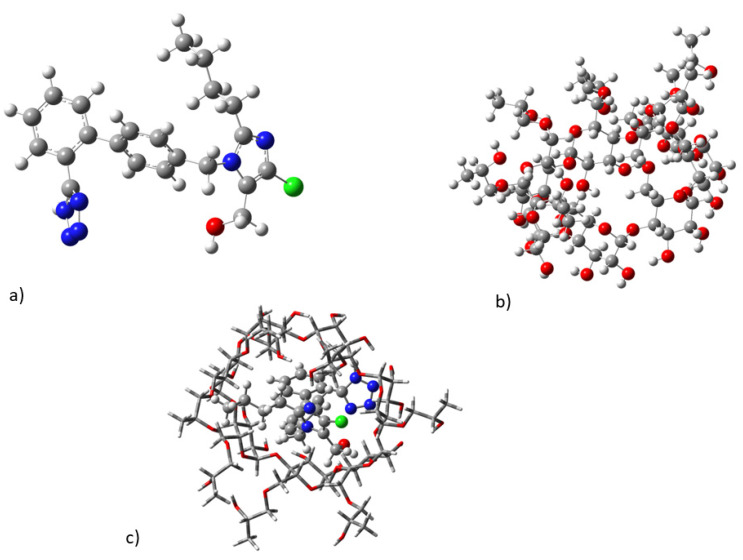
Calculated minimum energy structures: (**a**) losartan; (**b**) cyclodextrin; (**c**) losartan–cyclodextrin complex in water solvent at B3LYP/6-311G(d,p).

The DFT calculated lowest in energy minimum structures of losartan, 2HP-β-CD, and their complex in water solvent are depicted in Figure 7. Losartan inserts into 2HP-β-CD and its tetrazole group forms interactions with hydroxyl groups and protons 2 and 3. In addition, its butyl alkyl chain interacts with hydroxypropyl groups of 2HP-β-CD. The hydrophobic biphenyl ring is within the cavity of 2HP-β-CD, exerting hydrophobic interactions. It should be noted that we have initially inserted the losartan reversed inside cyclodextrin; however, this conformer of the complex was a local energy minimum lying 4.9 kcal/mol higher in energy than the complex in Figure 7c, and as a result it is not the favored one. Regarding the lowest in the energy conformer of the complex, see Figure 7c; it is in accordance with our experimental data and it explains the observed signals between the protons presented in Table 5.

Our calculated binding energies of the complex are given in Table 5. The values were also corrected for basis set superposition error (BSSE) BSSE results from the fact that the basis sets are not infinite. Thus, for the accurate description of the interaction energies and especially for non-covalent interactions, the BSSE should be considered [15]. We found that deformation energies, i.e., the energy difference between the structures of losartan and of 2HP-β-CD within the complex and the corresponding lowest in energy-free structures, are 4.8 and 10.6 kcal/mol, respectively. These values show that both species deformed significantly, especially the 2HP-β-CD, in order to increase the interaction. Note that the addition of the water solvent in calculation further stabilizes the CD, i.e., the deformation energy in gas is about 40% larger than in water. Moreover, our calculations show that the binding energy between the losartan and 2HP-β-CD is −5.47 kcal/mol (Table 7), i.e., it seems that the complex is stable. However, when the BSSE correction has been regarded, the complex is not stable by about 12.4 kcal/mol, i.e., losartan does not bind strongly in cyclodextrin, and as a result it can come in and out of the cyclodextrin. Finally, the binding energy with respect to the deformed structures of the molecules of the complex, BE_r_, is large enough; namely, −20.8 kcal/mol, showing that the complex can be isolated, and as a result it can be prepared for NMR experimentation. This energy characterizes the structural changes of the complex with respect to the isolated molecules.

## 3. Materials and Methods

### 3.1. Materials

Losartan (MW: 422.91) was kindly provided by Rafarm pharmaceutical company (Neo Psihiko, Athens, Greece). 2HP-β-CD (MW: 1460 g/mol) was purchased by Sigma-Aldrich (St Louis, MO, USA). The two molecules and their complexes were dissolved in deuterated water (5 mg/0.75 mL) purchased from Eurisotop, France.

### 3.2. Methods

#### 3.2.1. Sample Preparation

Lyophilized inclusion complex of LOS/HP-β-CD was prepared by freeze-drying aqueous solution of LOS/HP-β-CD using the neutralization method in molar ratio of 1:2, as described previously by Christodoulou et al. [16]. Briefly, 3445.6 mg of 2-HP-β-CD was accurately weighed and transferred into a glass vessel with 300 mL of purified water under magnetic stirring until complete dissolution of the cyclodextrin. Then, 500 mg of accurately weighed LOS potassium was added and dissolved under continuous stirring by adjusting the pH at approximately 10.5 with ammonium hydroxide solution 5% *v*/*v*. The volume of the obtained clear and colorless solution was fixed at 500 mL with purified water and was immediately frozen at −73 °C, and freeze-dried using Biobase Vacuum Freeze-Dryer, BK-FD10T, (Biobase Biodustry Co., Ltd., Shandong, China).

#### 3.2.2. Differential Scanning Calorimetry

The DSC thermograms of 2-HP-β-CD, losartan, their lyophilized forms, as well as their mixtures and complex were obtained with a DSC822^e^ Mettler-Toledo calorimeter (Schwerzenbach, Switzerland), after calibration with pure indium (T_m_ = 156.6 °C). 2-HP-β-CD and losartan were analyzed in their raw material and lyophilized forms. Then, all combinations between the two raw materials and lyophilized products were analyzed after mechanical mixing and grinding. Finally, the lyophilized complex between the two molecules was scanned. For each analysis, which was repeated three times, around 3 mg of dry powder of sample was weighed inside a 40 uL crucible, which was then sealed and left for a 15 min period to achieve equilibration. As we were aware that materials were critical for obtaining solid results, these were restrained from the same bottles. Afterwards, each analysis included a 5 min isotherm at 10 °C and a heating process from 10 °C to 300 °C, with a heating rate of 10 °C/min. The calorimetric data obtained (characteristic transition temperatures *T_onset_* and *T*, enthalpy change Δ*H* and width at half peak height of the *C_p_* profiles Δ*T_1/2_*) were analyzed using Mettler Toledo STAR^e^ software. The transition enthalpy was considered positive during an endothermic process.

#### 3.2.3. NMR Spectroscopy

The NMR (Nuclear Magnetic Resonance) characterization of the compounds was performed through standard 1D and 2D homo- and heteronuclear NMR experiments. For the NMR samples, 7 mg of losartan, 2HP-β-CD and their complex was diluted at 550 uL DMSO-d6. A set of NMR experiments was acquired for each sample with a relaxation delay (D1) of 1–2 s at room temperature (25 °C). In particular, a typical zg30 ^1^H-1D was performed using 30 degree flip angle with 64 scans. The Ernst angle of 30° is used for fast multi-scan experiments. Moreover, 2D homonuclear COSY (cosygpmfphpp) and ROESY (roesyadjsphpr) spectra were acquired with a spectral width of 10,504 Hz, 2048 and 256 data points in t2 and t1, respectively and 16 (ROESY) or 8 (COSY) scans. The relaxation delay of the homonuclear 2D spectra was set at 1.5 s. All spectra acquired with a Bruker Avance III HD 700 MHz spectrometer equipped with a 5 mm TCI cryoprobe and almost all pulse programs used are embedded in the standard Bruker library. Raw NMR data were processed with the standard Bruker NMR software (Topspin 3.5). Τ1 experiments were performed using the spin-echo pulse sequence installed in the library of the NMR spectrometer. The variable delay list contained ten different values for τ that were applied between 90° and 180°. The calculation of T1 for each proton was achieved using the MestReNova program. The equation of three parameters used was:

Β + F × exp(−xG)

where Β = magnetic induction field, F = spectral width and G = 1/T.

Degasification was not used in these experiments as the interest was the comparison between the T1 values and not the absolute ones. It is well known that typically, T1 values increase if the paramagnetic oxygen is removed through degasification.

#### 3.2.4. DFT Quantum Mechanics

The interaction of losartan with 2-hydroxypropyl-β-CD was calculated via the DFT methodology. At first, conformational analysis of losartan was carried out using the B3LYP [17,18]/6-311G(d,p) [19]) methodology to find the lowest in energy minimum structures [20]. Additionally, conformational analysis was carried out for the cyclodextrin and the complex of losartan–cyclodextrin via the semi-empirical PM6 methodology and then using the B3LYP/6-311G(d,p) methodology. All calculations were performed in water solvent employing the polarizable continuum model (PCM) [21]. Finally, in order to show how strongly losartan binds in the cyclodextrin, the interaction energy was calculated and this binding energy was corrected for the BSSE [22]. The calculations and the visualization were carried out via Gaussian 16 [23].

#### 3.2.5. Structures Preparation and Molecular Docking

**Figure 8:**
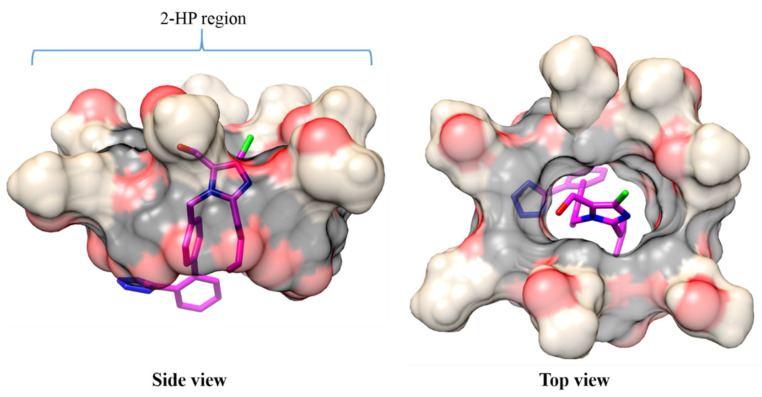
The optimal conformation of losartan into 2-HP-β-CD as obtained by molecular docking calculations. 2-HP-β-CD is depicted as transparent surface and the chlorine atom of losartan is in green. For simplicity, hydrogen atoms are not displayed.

The crystal structures of losartan and β-cyclodextrin (β-CD) were retrieved from the Cambridge Structural Database (CCDC and CSD reference codes: 253015 and BUVSEQ02, respectively). The 2-hydroxypropyl part was incorporated into β-CD to obtain the final structure of 2-HP-β-CD using Schrödinger 2015.2. [24] Losartan was docked inside 2-HP-β-CD with the chlorine end of the drug oriented toward the 2-HP edge of cyclodextrin (Figure 8) with the program UCSF DOCK6 [25]. The resulting losartan-2-HP-β-CD complex was then subjected to molecular dynamics (MD) simulations.

#### 3.2.6. Molecular Dynamics Simulations

The MD calculations were performed with the GPU version of PMEMD [26] from the AMBER16 [27] simulation software. The structure of losartan was geometrically optimized at the HF/6-31G * level of theory with Gaussian 09 [23]. The restrained electrostatic potential (RESP) approach [28,29] was applied to generate atomic charges for losartan, and the general AMBER force field (GAFF) was used to assign proper force-field parameters. GAFF was also employed for the treatment of the 2-hydroxypropyl groups of 2-HP-β-CD, while the cyclodextrin (β-CD) part was handled with the generalizable GLYCAM_06j-1 force field [30]. RESP charges were assigned to all atoms of the modified cyclodextrin. Next, the losartan–2-HP-β-CD complex was explicitly solvated in a truncated octahedral box containing ~3000 TIP3P water molecules [31]. The minimum distance between each solute atom and the edge of the periodic octahedron was set at 14 Å. The solvated complex was minimized for 30,000 total steps under constant volume (nonbonded cutoff = 10 Å). All solute atoms were initially fixed during 10,000 minimization cycles to allow for the relaxation of water molecules; constraints were gradually released for the next 10,000 cycles, and during the last part of the minimization (another 10,000 cycles), all atoms were freely moving. Then, the complex was gently heated from 0 to 310 K in two steps using a Langevin thermostat [32] under constant volume for 400 ps; a collision frequency (γ) of 2 ps^−1^ was applied. During the heating period, atomic positional restraints of 10 kcal mol^−1^ Å^−2^ limited the movement of the solute. Afterward, constant-pressure equilibration was conducted in two steps of 400 ps each. In the first step, the complex was restrained with a force constant of 10 kcal mol^−1^ Å^−2^ before it was allowed to freely move during the final step. Finally, an all-atom, unrestrained MD simulation for the 2-HP-β-CD complex with losartan in its docked conformation was carried out at 310 K, under constant pressure and for 500 ns. During the MD run, the SHAKE algorithm [33] was used to allow for a time-step of 2 fs by fixing all bond lengths involving hydrogen atoms close to their equilibrium distance.

*Trajectory Analysis and MM–PBSA/MM–GBSA Energy Calculations*: Conformational (distance, RMSD, RMS Fluctuation) and hydrogen bonding (HB) analyses of the resulting trajectories were performed with the *cpptraj* program [34] of AMBER. HB interactions were determined by cutoff definitions of 3.5 Å for the donor–acceptor distance and of 120° for the donor−hydrogen−acceptor angle. Binding free energy calculations were performed with the Molecular Mechanics Poisson–Boltzmann Surface Area (MM–PBSA) and the Molecular Mechanics Generalized Born Surface Area (MM–GBSA) methods [35,36,37]; details on the methodologies can be found in our previous publications [38,39,40,41].

## 4. Conclusions

The interactions of the prototype of sartan drug-molecules losartan with 2HP-β-CD were studied using a combination of DSC, NMR and in silico ΜD calculations. The thermal properties of lyophilized complex losartan:2HP-β-CD were compared with that of the mixture. The differences in their thermal properties allowed us to extract the conclusion that our procedure of complex preparation was confirmed. Intriguing differences between mixtures and lyophilized complexation also allowed the conclusion that thermal properties are highly affected by the procedure of the sample preparation.

NMR experiments clearly showed that complexation of losartan with 2HP-β-CD causes changes to chemical shifts, as was expected. These chemical shift changes drive conclusions on the way that losartan interacts with 2HP-β-CD. Application of the in silico MD calculations allowed us to quantify the distances between the interacting segments of losartan with those of 2HP-β-CD. Interestingly, the experimental and in silico methodologies were in harmony, and this confirms the way that losartan is inserted in the cavity of 2HP-β-CD to maximize its amphipathic interactions. Not only that, but this binding is reversible and thus provides excellent physical chemical properties to losartan to be delivered specifically to its site of action.

A previous pharmacological study by Paula et al. [6] that used ESI mass-spectrometry, NMR techniques, phase solubility and isothermal titration calorimetry investigated the water-soluble 1:1 complex of losartan with 2HP-β-CD. The authors concluded that this approach offers an alternative means of improving the bioavailability of water-soluble drugs and represents a significant step towards the development of sustained release of oral dosage forms. In addition, they claimed that LOS formulation accompanied by this strategy could be useful in reducing costs and increasing compliance with the antihypertensive treatment.

MD simulations and binding free energy calculations verified the favorable complexation between losartan and 2-HP-β-CD.

In agreement with the DFT-binding energy calculations (ΔGDFT = −5.5 kcal/mol), it was suggested that losartan has a sufficient affinity for 2-HP-β-CD (ΔGMM–PBSA: −4.8 kcal/mol, ΔGMM–GBSA: −6.7 kcal/mol, Table 1) that allows drug binding to occur (see references) [37,38,39].

While the phenyl–tetrazole group of losartan in the complex of cyclodextrin contains atoms with increased flexibility, its overall structure retains conformational stability through frequent hydrogen bonds with 2-HP-β-CD. MM–PB(GB)SA energy analysis in the complex suggested the suitability of cyclodextrin as a losartan carrier.

To conclude, our complementary study using additional NMR results, DSC experiments and quantitative in silico MD calculations supports and explains the findings of Paula et al. [6] and points out further experimental results using the same cyclodextrin, but also some others such the ones with methylated hydroxyl groups.

## Figures and Tables

**Table 1:** DSC thermodynamic parameters of the transition peaks of 2-HP-β-CD and losartan in raw material and lyophilized form, their mixtures and complex.

Sample	Molar Ratio	Weight Ratio	T_onset_ (°C)	T (°C)	ΔT_1/2_ (°C)	ΔH (J g^−1^)
**2-HP-β-CD Raw Material**	-	-	168.14	169.93	3.08	190.61
**2-HP-β-CD Lyophilized**	-	-	166.03	168.15	6.77	179.98
**Losartan** **Melting** **Pretransition**	-	-	267.70 231.91	271.77 238.59	4.10 6.22	117.16 10.23
**Losartan Lyophilized** **Melting** **Pretransition**	-	-	268.66 162.38	272.85 165.25	3.77 4.28	42.14 33.19
**Raw 2-HP-β-CD:Raw Losartan Mixture**	1:1	3.6:1	172.82	173.62	1.96	179.54
**Lyo 2-HP-β-CD:Raw Losartan Mixture**	1:1	3.6:1	175.79	178.04	5.06	202.20
**Raw 2-HP-β-CD:Lyo Losartan Mixture**	1:1	3.6:1	69.11	138.75	96.27	241.36
**Lyo 2-HP-β-CD:Lyo Losartan Mixture**	1:1	3.6:1	171.90	174.84	7.36	145.75
**2-HP-β-CD:Losartan Complex**	1:1	3.6:1	164.08	166.85	7.20	221.85

**Table 2:** In this table the chemical shift values of protons expressed in ppm of the three samples under study are shown; thus, losartan, 2HP-β-CD and the complex of losartan:2HP-β-CD.

Position	Losartan	Complex of Losartan: 2HP-β-CD
	1H (ppm)	1H (ppm)
10	0.61	0.72
9	1.02	1.17
8	1.28	1.42
7	2.33	2.52
6	4.26	4.41
11	5.01	5.18
13–17	6.64	6.88–6.87
14–16	6.79	7.06–7.05
19	7.12	7.42
21	7.34	7.50
20	7.36	7.51
22	7.49	7.56
Position	2HPβCD	Complex of losartan: 2HP-β-CD
	1H (ppm)	1H (ppm)
9	1.07	1.07
2, 4, 5, 7	3.38–3.60	3.37–3.58
3	3.65	3.65
6	3.80	3.78
3, 6	3.85–3.90	3.81
8	3.95	3.93
1	5.01	4.98
1′	5.18	5.01

**Table 3:** The most important comparative distances in the complex obtained through 2D ROESY and DFT MD calculations.

Spatial Interactions	Distances Expressed in Å Obtained through MD Calculations	Qualitative Characterization of the Spatial Interactions
H10 (LOSARTAN)-H6(2-HΡ-β-CD)	5.33	w
H10 (LOSARTAN)-H8(2-HP-β-CD)	5.18	w
H10 (LOSARTAN)-H7(2-HP-β-CD)	5.22	w
H13 (LOSARTAN)-H3(2-HP-β-CD)	4.70	w
H17 (LOSARTAN)-H1(2-HP-β-CD)	5.02	w
H17(LOSARTAN)-H3(2-HP-β-CD)	4.19	m
H16(LOSARTAN)-H3(2-HP-β-CD)	2.64	s
H14(LOSARTAN)-H1(2-HP-β-CD)	4.91	w
H14(LOSARTAN)-H3(2-HP-β-CD)	2.98	s
H19(LOSARTAN)-H3(2-HP-β-CD)	2.57	s
H19(LOSARTAN)-H6(2-HP-β-CD)	4.55	w
H19(LOSARTAN)-H1(2-HP-β-CD)	4.82	w
H19(LOSARTAN)-H2(2-HP-β-CD)	5.20	w
H20(LOSARTAN)-H3(2-HP-β-CD)	3.88	m
H20(LOSARTAN)-H2(2-HP-β-CD)	4.31	m
H21(LOSARTAN)-H3(2-HP-β-CD)	4.25	m
H22(LOSARTAN)-H3(2-HP-β-CD)	5.22	w
H11(LOSARTAN)-H7(2-HP-β-CD)	5.01	w
H11(LOSARTAN)-H6(2-HP-β-CD)	4.08	m
H11(LOSARTAN)-H5(2-HP-β-CD)	3.25	s
H6(LOSARTAN)-H9(2-HP-β-CD)	4.57	w
H6(LOSARTAN)-H8(2-HP-β-CD)	4.55	w
H6(LOSARTAN)-H6(2-HP-β-CD)	3.66	m
H6(LOSARTAN)-H5(2-HP-β-CD)	3.72	m
H7(LOSARTAN)-H6(2-HP-β-CD)	3.73	m
H7(LOSARTAN)-H7(2-HP-β-CD)	4.46	m
H7(LOSARTAN)-H5(2-HP-β-CD)	4.07	m

s = strong; m = medium; w = weak.

**Table 4:** In this table the T1 values of protons of the three samples under study are shown; thus, losartan, 2HP-β-CD and the complex of losartan:2HP-β-CD.

**2-hp-β-CD**			
**Free 2HP-β-CD**		**Complexed 2HP-β-CD**	
**Chemical Shifts (ppm)**	**T1 (s)**	**Chemical Shifts**	**T1 (s)**
1.07 (9)	0.63	1.07 (9)	0.59
3.80 (6)	0.66	3.78 (6)	0.54
3.95 (8)	1.10	3.93	0.76
5.01 (1)	0.94	4.98	0.71
**Losartan**			
**Free Losartan**		**Complexed Losartan**	
**Chemical Shifts**	**T1 (s)**	**Chemical Shifts**	**T1 (s)**
0.61 (10)	0.65	0.72 (10)	1.19
1.02 (9)	0.58	1.17 (9)	0.52
1.28 (8)	0.53	1.42 (8)	0.48
2.33 (7)	0.47	2.52 (7)	
4.26 (6)	0.44	4.41 (6)	0.47
5.01 (11)	0.40	5.18 (11)	0.54
6.64 (13/17)	0.60	6.88–6.87 (13/17)	0.79
6.79 (14/16)	0.66	7.06–7.05 (14/16)	0.84
7.12 (19)	0.62	7.42 (19)	0.83
7.34 (21)	0.66	7.50 (21)	0.93
7.36 (20)	0.67	7.51 (20)	0.94
7.49 (22)	0.82	7.56 (22)	1.02

**Table 5:** The most important spatial correlations for losartan.

Spatial Interactions	Distances Expressed in Å	Qualitative Characterization of the Spatial Interactions
H10-H13/H17	5.20	w
H9-H13/H17	3.78	m
H8-H13/H17	3.70–3.77	m
H7-H13/H17	4.64	w
H6-H11	2.19–3.67	s
H11-H7	2.28 (3.75)	s
H8-H11	3.92	m
H7-H14	4.74	w
H7-H16	5.88	w
H7-H19	5.84	w
H14-H19	2.65	s

s = strong, m = medium, w = weak.

**Table 6:** MM–PBSA and MM–GBSA free energy calculations for the losartan–2-HP-β-CD complex.

Energy (kcal mol^−1^)	MM–PBSA	MM–GBSA
Δ*E*_vdW_	−33.19 ± 0.09 *^a^*	−33.19 ± 0.09
Δ*E*_elec_	−5.30 ± 0.25	−5.30 ± 0.25
Δ*E*_MM, gas_	−38.49 ± 0.28	−38.49 ± 0.28
Δ*G*_PB/GB_	15.53 ± 0.18	14.53 ± 0.20
Δ*G*_elec(tot)_ *^b^*	10.23 ± 0.26	9.23 ± 0.13
Δ*G*_NP_	−3.00 ± 0.00	−3.88 ± 0.01
Δ*G*_solv_	12.52 ± 0.18	10.67 ± 0.20
**Δ*H*_(MM + solv)_**	−25.97 ± 0.13	−27.83 ± 0.11
**−*T*Δ*S*_tot_**	21.15 ± 0.12	21.15 ± 0.12
**Δ*G*_MM–PB(GB)SA_**	−4.82 ± 0.13 *^c^*	−6.68 ± 0.11 *^c^*

*^a^* Standard errors of the mean (SEM): SEM = standard deviation/√N, where N equals the number of trajectory frames used in MM−PB (GB) S A calculations (500 for entropy, and 4000 for everything else). *^b^*Δ*G*elec_(tot)_ = Δ*E*_elec_ + Δ*G*_PB/GB_. *^c^* The errors for Δ*G*_MM–PB(GB)SA_ have been estimated as pooled SEM based on the following formula: $S=\sqrt{\frac{\left(n_{1}-1\right){\left(s_{1}\right)}^{2}+\left(n_{2}-1\right){\left(s_{2}\right)}^{2}}{n_{1}+n_{2}-2}}$, where *n*_1_ is the number of frames for the enthalpy (Δ*H*) calculation (*n*_1_ = 4000), *n*_2_ is the number of frames for the entropy (−*T*Δ*S*) calculation (*n*_2_ = 500) and *s*_1_ and *s*_2_ are the SEM of Δ*H* and −*T*Δ*S* calculations, respectively.

**Table 7:** Binding energies, BE; corrected values for BSSE, BE_BSSE_, and binding energies with respect to the deformed structures of the molecules of the complex, BE_r_ (kcal/mol); deformation energies of the losartan and cyclodextrin, Def_L_ and Def_CD_, at PM6 and B3LYP/6-311G(d,p). All energies are in kcal/mol.

	PM6 ^a^	B3LYP ^b^	B3LYP ^a^
BE	−3.06	−6.61	−5.47
BE_BSSE_	---	12.40	12.10
BE_r_	−12.21	−28.53	−20.85
Def_L_	−4.38	5.83	4.76
Def_CD_	---	16.09	10.60

^a^ In water solvent; ^b^ in that gas phase.

## Data Availability

Available upon request.

## Supplementary Materials

The following supporting information can be downloaded at: https://www.mdpi.com/article/10.3390/molecules27082421/s1, Figure S1: 2D COSY NMR spectra of losartan and 2-hp-β-CD complex obtained with Bruker spectrometer using D_2_O solvent and at 25 °C, Figure S2: 2D COSY NMR spectra of losartan with D2O solvent and at 25 °C, Figure S3: 2D COSY NMR spectra of losartan obtained with Bruker spectrometer using D2O solvent and at 25 °C, Figure S4: Relaxation time of losartan and 2-hp-β-CD complex; Figure S5. Relaxation time of losartan, Figure S6: Relaxation time of 2-hp-β-CD, Figure S7: Root mean square deviations (RMSD) of cyclodextrin (2-HP-β-CD) and losartan in the inclusion complex.

## Sample Availability

Samples of the compounds are available from the authors.

## References

[1] Kellici T.F., Tzakos A.G., Mavromoustakos T. (2015). Rational Drug Design and Synthesis of Molecules Targeting the Angiotensin II Type 1 and Type 2 Receptors. Molecules.

[2] Kellici T.F., Liapakis G., Tzakos A.G., Mavromoustakos T. (2015). Pharmaceutical Compositions for Antihypertensive Treatments: A Patent Review. Expert Opin. Ther. Pat..

[3] Nejat R., Sadr A.S. (2021). Are losartan and imatinib effective against SARS-CoV2 pathogenesis? A pathophysiologic-based in silico study. Silico Pharmacol..

[4] Chroni A., Mavromoustakos T., Pispas S. (2021). Nano-Assemblies from Amphiphilic PnBA-b-POEGA Copolymers as Drug Nanocarriers. Polymers.

[5] Chroni A., Mavromoustakos T., Pispas S. (2020). Biocompatible PEO-b-PCL Nanosized Micelles as Drug Carriers: Structure and Drug–Polymer Interactions. Nanomaterials.

[6] De Paula W.X., Denadai M.L., Santoro M.M., Braga A.N.G., Santos R.A.S., Sinisterra R.D. (2011). Supramolecular interactions between losartan and hydroxypropyl-β-CD: ESI mass-spectrometry, NMR techniques, phase solubility, isothermal titration calorimetry and anti-hypertensive studies. Int. J. Pharm..

[7] De Melo C.M., Da Silva A.L., de Melo K.R., Da Silva P.C.D., de Souza M.L., de Sousa A.L.M.D., Rabello M.M., Véras L.M.C., Rolim L.A., Neto P.J.R. (2021). In silico and in vitro study of epiisopiloturine/ hydroxypropyl-β-cyclodextrin inclusion complexes obtained by different methods. J. Drug Deliv. Sci. Technol..

[8] Mura P. (2015). Analytical techniques for characterization of cyclodextrin complexes in the solid state: A review. J. Pharm. Biomed. Anal..

[9] Kratz J.M., Teixeira M.R., Ferronato K., Teixeira H.F., Koester L.S., Simões C.M.O. (2012). Preparation, Characterization, and In Vitro Intestinal Permeability Evaluation of Thalidomide–Hydroxypropyl-β-Cyclodextrin Complexes. AAPS PharmSciTech.

[10] Talegaonkar S., Yakoob Khan A., Kishan Khar R., Jalees Ahmad F., Khan Z. (2010). Development and Characterization of Paracetamol Complexes with Hydroxypropyl-?-Cyclodextrin. Iran. J. Pharm. Res..

[11] Da Silva L.F.J.S., do Carmo F.A., de Almeida Borges V.R., Monteiro L.M., Rodrigues C.R., Cabral L.M., de Sousa V.P. (2011). Preparation and Evaluation of Lidocaine Hydrochloride in Cyclodextrin Inclusion Complexes for Development of Stable Gel in Association with Chlorhexidine Gluconate for Urogenital Use. Int. J. Nanomed..

[12] Khandai M., Chakraborty S., Ghosh A.K. (2014). Losartan Potassium Loaded Bioadhesive Micro-Matrix System: An Investigation on Effects of Hydrophilic Polymeric Blend on Drug Release. Pharm. Anal. Acta.

[13] Liu M., Cao W., Sun Y., He Z. (2014). Preparation, characterization and in vivo evaluation of formulation of repaglinide with hydroxypropyl-β-cyclodextrin. Int. J. Pharm..

[14] De Freitas M.R., Rolim L.A., Soares M.F.D.L.R., Rolim-Neto P.J., de Albuquerque M.M., Soares-Sobrinho J.L. (2012). Inclusion complex of methyl-β-cyclodextrin and olanzapine as potential drug delivery system for schizophrenia. Carbohydr. Polym..

[15] Van Duijneveldt F.B., van Duijneveldt-van de Rijdt J.G., van Lenthe J.H. (1994). State of the Art in Counterpoise Theory. Chem. Rev..

[16] Christodoulou E., Ntountaniotis D., Leonis G., Mavromoustakos T., Valsami G. (2021). Application of Neutralization and Technique for the Preparation of the Beneficial in Drug 2-Hydroxypropyl-β-Cyclodextrin with. Supramolecules in Drug Discovery and Drug Delivery.

[17] Lee C., Yang W., Parr R.G. (1988). Development of the Colle-Salvetti correlation-energy formula into a functional of the electron density. Phys. Rev. B.

[18] Becke A.D. (1993). A new mixing of Hartree–Fock and local density-functional theories. J. Chem. Phys..

[19] Curtiss L.A., McGrath M.P., Blaudeau J., Davis N.E., Binning R.C., Radom L. (1995). Extension of Gaussian-2 theory to molecules containing third-row atoms Ga–Kr. J. Chem. Phys..

[20] Tzeli D., Tsoungas P.G., Petsalakis I.D., Kozielewicz P., Zloh M. (2015). Intramolecular cyclization of β-nitroso-o-quinone methides. A theoretical endoscopy of a potentially useful innate ‘reclusive’ reaction. Tetrahedron.

[21] Cossi M., Scalmani G., Rega N., Barone V. (2002). New developments in the polarizable continuum model for quantum mechanical and classical calculations on molecules in solution. J. Chem. Phys..

[22] Tzeli D., Mavridis A., Xantheas S.S. (2002). First Principles Examination of the Acetylene−Water Clusters, HCCH−(H2O)x, x = 2, 3, and 4. J. Phys. Chem. A.

[23] Frisch M.J., Trucks G.W., Schlegel H.B., Scuseria G.E., Robb M.A., Cheeseman J.R., Scalmani G., Barone V., Petersson G.A., Nakatsuji H. (2009). Gaussian 16, Revision B.01. Gaussian 09.

[24] Schrödinger (2015). Schrödinger Release 2015-2.

[25] Lang P.T., Brozell S.R., Mukherjee S., Pettersen E.F., Meng E.C., Thomas V., Rizzo R.C., Case D.A., James T.L., Kuntz I.D. (2009). DOCK 6: Combining techniques to model RNA–small molecule complexes. RNA.

[26] Salomon-Ferrer R., Götz A.W., Poole D., Le Grand S., Walker R.C. (2013). Routine Microsecond Molecular Dynamics Simulations with AMBER on GPUs. 2. Explicit Solvent Particle Mesh Ewald. J. Chem. Theory Comput..

[27] Case D.A., Berryman J., Betz R.M., Cerutti D.S., Cheatham Iii T.E., Darden T.A., Duke R.E., Giese T.J., Gohlke H., Goetz A.W. (2016). Amber 2016.

[28] Bayly C.I., Cieplak P., Cornell W., Kollman P.A. (1993). A well-behaved electrostatic potential based method using charge restraints for deriving atomic charges: The RESP model. J. Phys. Chem..

[29] Wang J., Wolf R.M., Caldwell J.W., Kollman P.A., Case D.A. (2004). Development and testing of a general amber force field. J. Comput. Chem..

[30] Kirschner K.N., Yongye A.B., Tschampel S.M., González-Outeiriño J., Daniels C.R., Foley B.L., Woods R.J. (2008). GLYCAM06: A generalizable biomolecular force field. Carbohydrates. J. Comput. Chem..

[31] Jorgensen W.L., Chandrasekhar J., Madura J.D., Impey R.W., Klein M.L. (1983). Comparison of simple potential functions for simulating liquid water. J. Chem. Phys..

[32] Izaguirre J.A., Catarello D.P., Wozniak J.M., Skeel R.D. (2001). Langevin stabilization of molecular dynamics. J. Chem. Phys..

[33] Ryckaert J.-P., Ciccotti G., Berendsen H.J.C. (1977). Numerical integration of the cartesian equations of motion of a system with constraints: Molecular dynamics of n-alkanes. J. Comput. Phys..

[34] Roe D.R., Cheatham T.E. (2013). PTRAJ and CPPTRAJ: Software for Processing and Analysis of Molecular Dynamics Trajectory Data. J. Chem. Theory Comput..

[35] Kollman P.A., Massova I., Reyes C., Kuhn B., Huo S., Chong L., Lee M., Lee T., Duan Y., Wang W. (2000). Calculating Structures and Free Energies of Complex Molecules: Combining Molecular Mechanics and Continuum Models. Accounts Chem. Res..

[36] Gohlke H., Kiel C., Case D.A. (2003). Insights into Protein–Protein Binding by Binding Free Energy Calculation and Free Energy Decomposition for the Ras–Raf and Ras–RalGDS Complexes. J. Mol. Biol..

[37] Wang W., Kollman P.A. (2001). Computational study of protein specificity: The molecular basis of HIV-1 protease drug resistance. Proc. Natl. Acad. Sci. USA.

[38] Kellici T.F., Chatziathanasiadou M.V., Diamantis D., Chatzikonstantinou A.V., Andreadelis I., Christodoulou E., Valsami G., Mavromoustakos T., Tzakos A.G. (2016). Mapping the Interactions and Bioactivity of Quercetin-(2-Hydroxypropyl)-β-Cyclodextrin Complex. Int. J. Pharm..

[39] Kellici T.F., Ntountaniotis D., Leonis G., Chatziathanasiadou M., Chatzikonstantinou A.V., Becker-Baldus J., Glaubitz C., Tzakos A.G., Viras K., Chatzigeorgiou P. (2015). Investigation of the Interactions of Silibinin with 2-Hydroxypropyl-β-cyclodextrin through Biophysical Techniques and Computational Methods. Mol. Pharm..

[40] Leonis G., Christodoulou E., Ntountaniotis D., Chatziathanasiadou M.V., Mavromoustakos T., Naziris N., Chountoulesi M., Demetzos C., Valsami G., Damalas D.E. (2020). Antihypertensive activity and molecular interactions of irbesartan in complex with 2-hydroxypropyl-β-cyclodextrin. Chem. Biol. Drug Des..

[41] Tzoupis H., Leonis G., Mavromoustakos T., Papadopoulos M.G. (2013). A Comparative Molecular Dynamics, MM–PBSA and Thermodynamic Integration Study of Saquinavir Complexes with Wild-Type HIV-1 PR and L10I, G48V, L63P, A71V, G73S, V82A and I84V Single Mutants. J. Chem. Theory Comput..

