# Supplementary material for: Losartan Interactions with 2-Hydroxypropyl-β-CD
Source: Molecules. 2022 Apr 8;27(8):2421. doi: 10.3390/molecules27082421 (PMC9027256; doi:10.3390/molecules27082421)
Supplement: Supplementary file 1 [file molecules-27-02421-s001.zip › molecules-1651831-supplementary.pdf]

SUPPLEMENTARY MATERIAL

Losartan interactions with 2-hydroxypropyl-β-CD

Vasiliki Palli [1], Georgios Leonis [1], Nikoletta Zoupanou [1], Nikitas Georgiou [1], Maria Chountoulesi [2], Nikolaos Naziris [2,3], Demeter Tzeli [1,4], Costas Demetzos [2], Georgia Valsami [2,*], Konstantinos D. Marousis [5], Georgios A. Spyroulias [5] and Thomas Mavromoustakos [1,*]

[1]Department of Chemistry, School of Sciences, National and Kapodistrian University of Athens, Zografou,15784, Greece
[2]Department of Pharmacy, School of Health, Sciences, National and Kapodistrian University of Athens, Zografou 15784, Greece
[3]Department of General Biophysics, Faculty of Biology and Environmental Protection, University of Lodz, Pomorska 141/143, Lodz 90-236, Poland
Department of Pharmacy, University of Patras, 26504 Patras, Greece
[4]Theoretical and Physical Chemistry Institute, National Hellenic Research Foundation, 48 Vassileos Constantinou Ave., Athens 116 35, Greece
[5] Department of Pharmacy, University of Patras, 26504 Patras, Greece

*Correspondence: tmavrom@chem.uoa.gr; valsami@pharm.uoa.gr

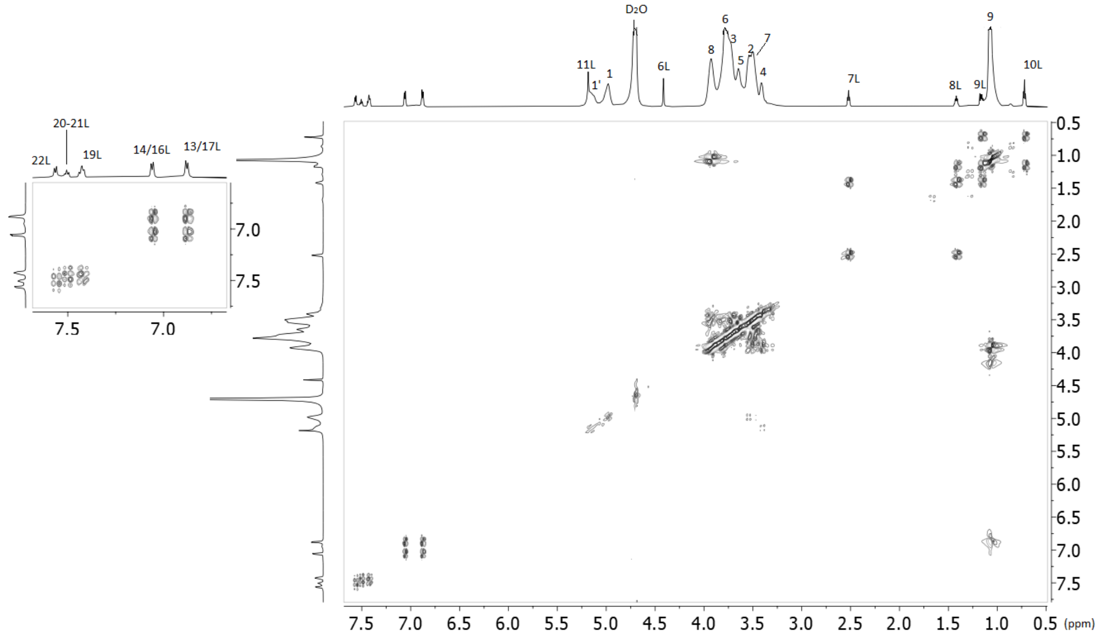

Figure S1. 2D COSY NMR spectra of losartan and 2-hp-β-CD complex obtained with 700 MHz Bruker spectrometer using D_2O solvent and at 25 °C.

Figure S2. 2D COSY NMR spectra of losartan with 700 MHz Bruker spectrometer using D2O solvent and at 25^0C

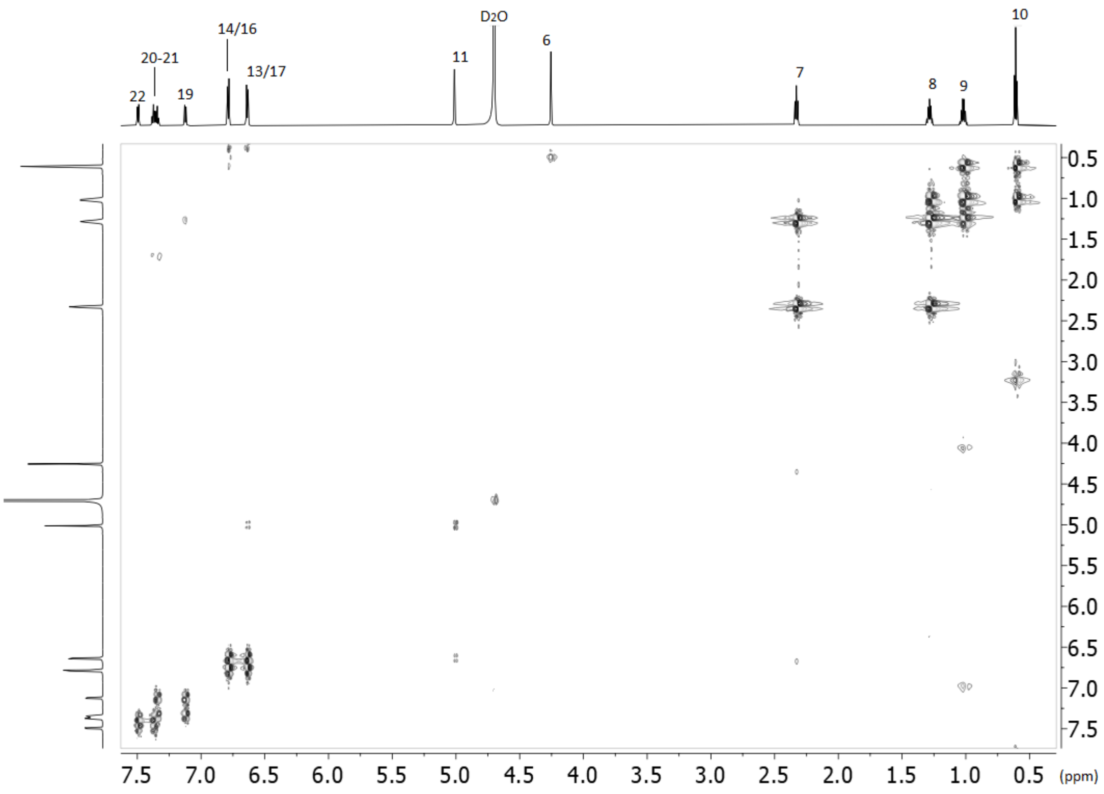

Figure S2. 2D COSY NMR spectra of losartan with 700 MHz Bruker spectrometer using D2O solvent and at 25 °C.

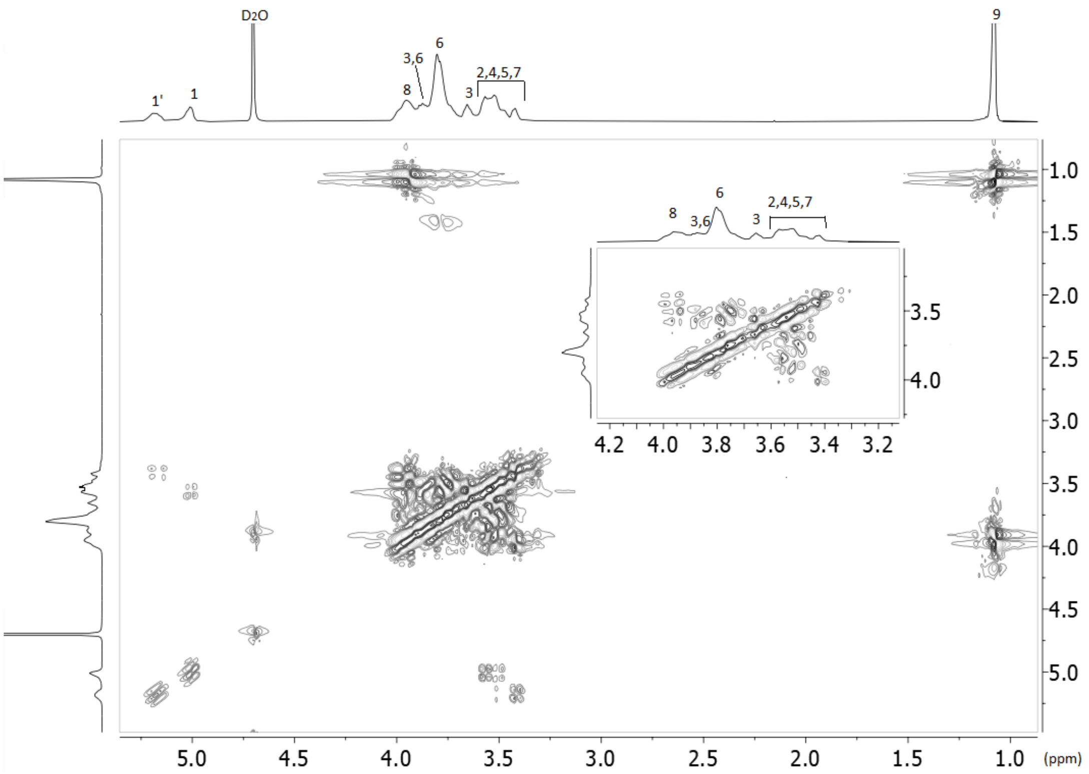

Figure S3. 2D COSY NMR spectra of losartan obtained with 700 MHz Bruker spectrometer using D2O solvent and at 25 °C.

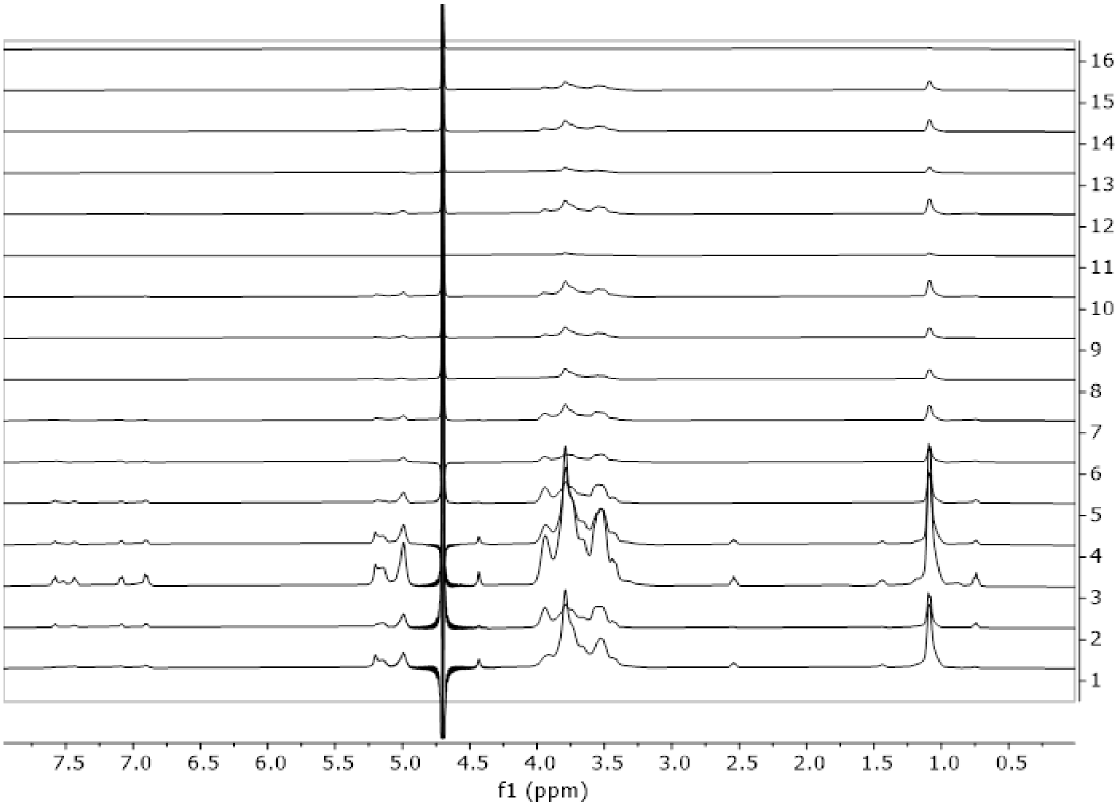

Figure S4. Relaxation time of losartan and 2-hp-β-CD complex.

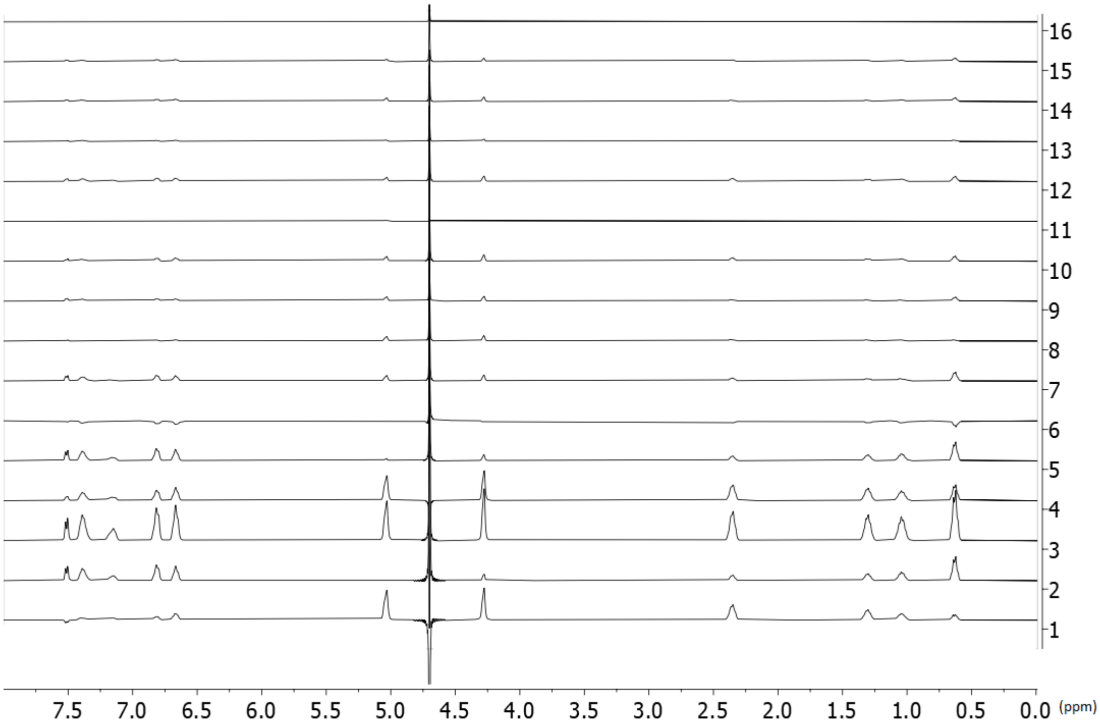

Figure S5. Relaxation time of losartan.

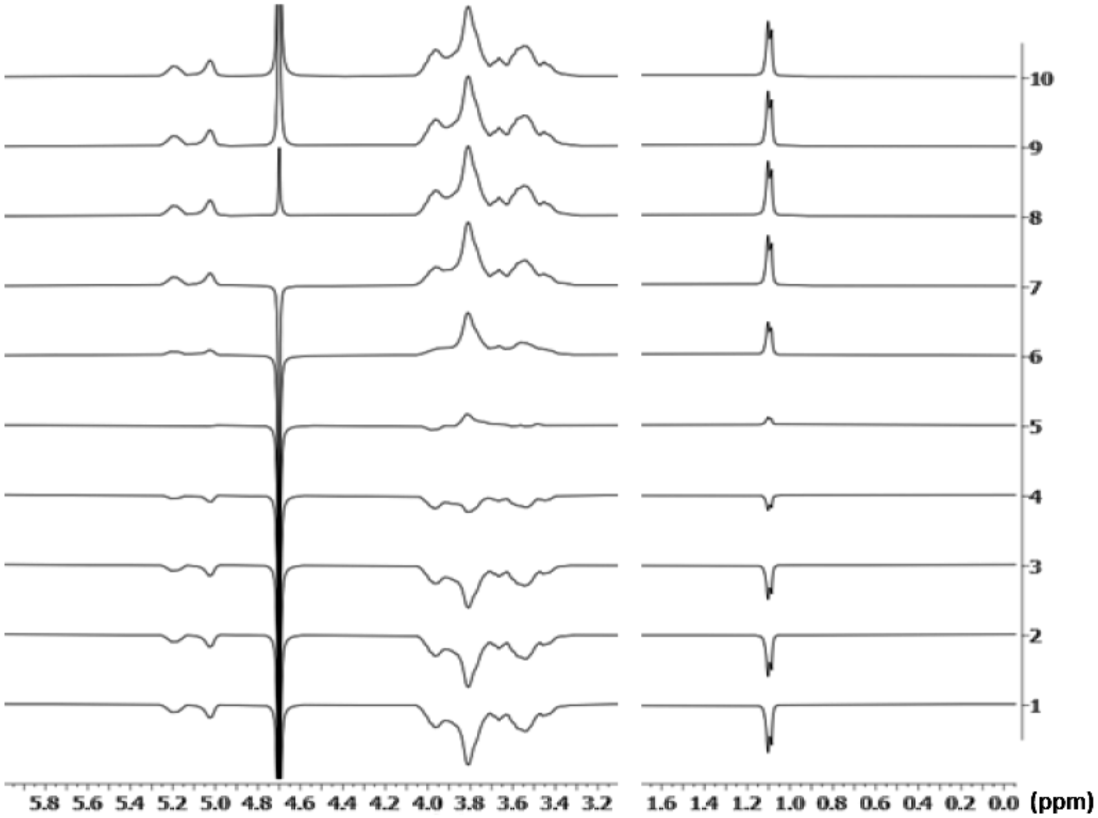

Figure S6. Relaxation time of 2-hp-β-CD.

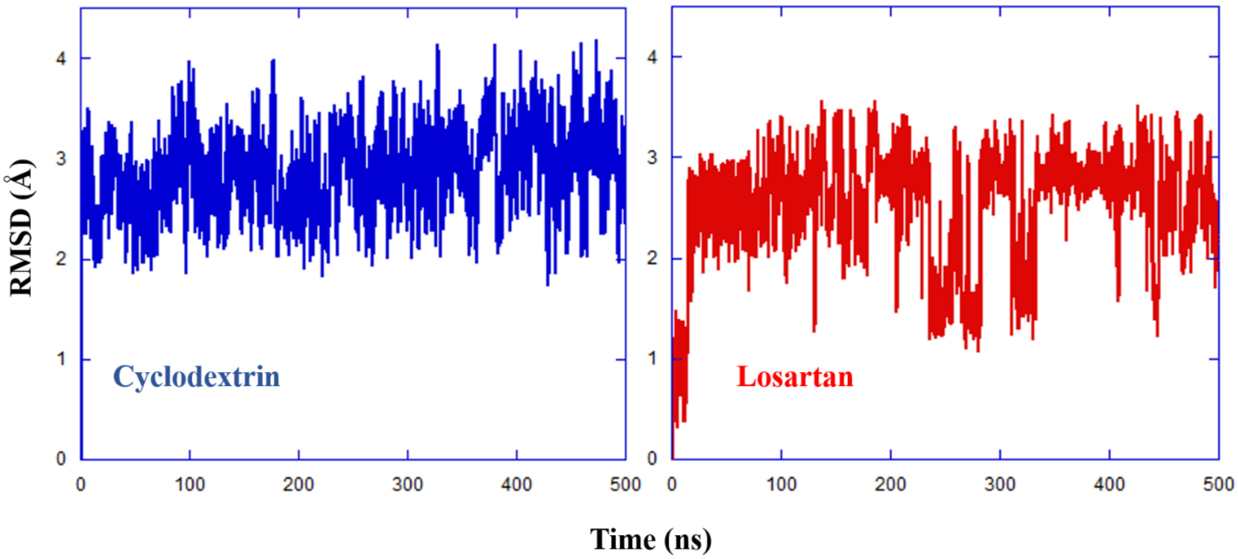

Figure S7. Root mean square deviations (RMSD) of cyclodextrin (2-HP-β-CD) and losartan in the inclusion complex.
